# Association between Single Nucleotide Polymorphism rs1044925 and the Risk of Coronary Artery Disease and Ischemic Stroke

**DOI:** 10.3390/ijms15033546

**Published:** 2014-02-26

**Authors:** Dong-Feng Wu, Rui-Xing Yin, Xiao-Li Cao, Wu-Xian Chen

**Affiliations:** 1 Department of Cardiology, Institute of Cardiovascular Diseases, the First Affiliated Hospital, Guangxi Medical University, 22 Shuangyong Road, Nanning 530021, Guangxi, China; E-Mails: wulove26@tom.com (D.-F.W.); nncwx@163.com (W.-X.C.); 2 Department of Neurology, the First Affiliated Hospital, Guangxi Medical University, 22 Shuangyong Road, Nanning 530021, Guangxi, China; E-Mail: maten1996@gmail.com

**Keywords:** acyl-CoA:cholesterol acyltransferase gene, single nucleotide polymorphism, coronary artery disease, ischemic stroke, lipids

## Abstract

The present study was performed to clarify the association between the acyl-CoA:cholesterol acyltransferase-1 (*ACAT-1*) single nucleotide polymorphism (SNP) rs1044925 and the risk of coronary artery disease (CAD) and ischemic stroke (IS) in the Guangxi Han population. Polymerase chain reaction and restriction fragment length polymorphism was performed to determine the genotypes of the *ACAT-1* SNP rs1044925 in 1730 unrelated subjects (CAD, 587; IS, 555; and healthy controls; 588). The genotypic and allelic frequencies of rs1044925 were significantly different between the CAD patients and controls (*p* = 0.015) and borderline different between the IS patients and controls (*p* = 0.05). The AC/CC genotypes and C allele were associated with a decreased risk of CAD and IS (CAD: *p* = 0.014 for AC/CC *vs*. AA, *p* = 0.022 for C *vs*. A; IS: *p* = 0.014 for AC/CC *vs*. AA; *p* = 0.017 for C *vs*. A). The AC/CC genotypes in the healthy controls, but not in CAD or IS patients, were associated with an increased serum high-density lipoprotein cholesterol (HDL-C) concentration. The present study shows that the C allele carriers of *ACAT-1* rs1044925 were associated with an increased serum HDL-C level in the healthy controls and decreased risk in CAD and IS patients.

## 1. Introduction

Cholesterol is present in the membranes of all mammalian cells and is needed for their growth and viability. Excess cellular cholesterol is stored as cholesteryl esters (CEs). In most cell types, CEs are present only in low levels, mainly as cytoplasmic lipid droplets. Chronic accumulation of CE in macrophages causes these cells to appear foamy and is a hallmark of early-stage atherosclerosis [1]. The formation of CEs is catalyzed by the enzyme acyl-coenzyme A (CoA):cholesterol acyltransferase (ACAT) [2]. There are two isozymes of ACAT, ACAT-1 and ACAT-2, with different intracellular localization, membrane topology in mammalian species, and metabolic function for each enzyme [3–5]. ACAT-1 is ubiquitously expressed in various tissues and cells, including the brain, adrenal glands, kidneys [6–8], and macrophages [9], and is responsible for foam cell formation in macrophages, whereas ACAT-2 is expressed only in the intestines and liver [3,4,10] and regulates cholesterol absorption in intestinal mucosal cells [11]. Therefore, pharmacological inhibition of ACAT is expected to suppress foam cell formation in arterial walls by suppressing macrophage ACAT-1 and cholesterol absorption by suppressing intestinal ACAT-2, thereby inhibiting atherosclerosis [12].

A number of animal studies have suggested that ACAT inhibitors might be promising drugs for controlling hyperlipidemia and atherosclerosis [12,13]. However, a drug that inhibits ACAT activity could cause a mild increase in low-density lipoprotein cholesterol (LDL-C) and in the incidence of major cardiovascular events in humans [14–16]. For an isoform of ACAT, ACAT-2 has been proven to be a protective factor in atherosclerosis in animal models [17,18]. Although, whether attenuating ACAT-1 can improve atherosclerosis is under debate, Yagyu *et al*. [19] showed that ACAT-1 inhibition in tissue macrophages is protective against CE accumulation, thereby attenuating atherosclerosis. However, in LDL receptor- or apoE-knockout mice, selective deficiency of the ACAT-1 isoform resulted in larger atherosclerotic lesions [20]. Further studies in hyperlipidemia models showed that the detrimental effects of *ACAT-1* deficiency were attributed to increased macrophage apoptosis caused by a massive accumulation of free cholesterol and impaired cellular cholesterol efflux [21–23]. Therefore, the role of *ACAT-1* in atherosclerosis remains to be clarified.

To our knowledge, the genetic evidence on the association between *ACAT-1* variants and atherosclerosis in humans is poor. In a previous study, we have found that the *ACAT-1* SNP rs1044925 modulated the serum high-density lipoprotein cholesterol (HDL-C) concentration in the hypercholesterolemic subjects [24], suggesting that rs1044925 influences the cellular cholesterol efflux in hypercholesterolemia and plays an important role in the formation of atherosclerosis. Therefore, the present study aimed to determine whether the *ACAT-1* SNP rs1044925 is associated with the risk of coronary artery disease (CAD) and ischemic stroke (IS).

## 2. Results

### 2.1. General Characteristics and Serum Lipid Levels

The baseline characteristics of the patients with CAD or IS and the controls are shown in Table 1. The mean age, male to female ratio, serum LDL-C and apolipoprotein (Apo) B levels and the percentages of subjects who smoked were similar between the controls and CAD patients and between the controls and IS patients. The average body mass index (BMI), diastolic blood pressure, pulse pressure, and serum triglyceride (TG) levels were significantly higher and serum total cholesterol (TC), HDL-C, Apo AI, ApoAI/ApoB ratio and the percentages of subjects who consumed alcohol were significantly lower in the CAD and IS patients than in the controls.

### 2.2. Genotypic and Allelic Frequencies

The frequency of the A and C alleles was 84.2% and 15.8% in the controls, 87.5% and 12.5% in the CAD patients, and 87.7% and 12.3% in the IS patients respectively (Table 2). The frequency of the AA, AC and CC genotypes was 70.1%, 28.2% and 1.7% in the controls, 77.0%, 21.0% and 2.0% in the CAD patients, and 76.4%, 22.5% and 1.1% in the IS patients respectively. The genotypic and allelic frequencies were different between the control and CAD patients and between the controls and IS patients. The genotypic and allelic frequencies were concordant with those predicted by the Hardy-Weinberg proportions in both experimental groups (*p* = 0.292 for CAD and *p* = 0.336 for IS) and controls (*p* = 0.145).

### 2.3. *ACAT-1* SNP rs1044925 and the Risk of CAD and IS

The C allele was associated with a decreased risk of CAD (adjusted Odds ratio (OR) = 0.76, 95% confidence interval (CI) = 0.60–0.96) and IS (adjusted OR = 0.75, 95% CI = 0.59–0.95) (Table 2). The AC and AC/CC genotypes were also associated with a decreased risk of CAD (adjusted OR = 0.67, 95% CI = 0.50–0.91 for AC *vs*. AA and adjusted OR = 0.69, 95% CI = 0.52–0.93 for AC/CC *vs*. AA) and IS (adjusted OR = 0.72, 95% CI = 0.54–0.95 for AC *vs*. AA and adjusted OR = 0.71, 95% CI = 0.54–0.93 for AC/CC *vs*. AA). Stratified analysis showed a decreased risk of CAD in subjects with an AC/CC genotype, mainly in those who belonged to one of the following groups: high BMI (adjusted OR = 0.57, 95% CI = 0.36–0.91), males (adjusted OR = 0.71, 95% CI = 0.50–1.00), nonsmokers (adjusted OR = 0.63, 95% CI = 0.42–0.94) and nondrinkers (adjusted OR = 0.66, 95% CI = 0.47–0.93). There was a decreased risk of IS in subjects with an AC/CC genotype, mainly in those who belonged to one of the following groups: high BMI (adjusted OR = 0.53, 95% CI = 0.34–0.84), males (adjusted OR = 0.69, 95% CI = 0.50–0.95) and nondrinkers (adjusted OR = 0.68, 95% CI = 0.49–0.95 Table 3). No significant interaction was detected between the genotypes and these factors.

### 2.4. Related Risk Factors for CAD and IS

Multivariate logistic analysis showed that the incidence of CAD and IS positively correlated with BMI, diabetes, hypertension and hyperlipidemia and negatively correlated with the rs1044925 AC/CC genotypes and alcohol consumption (Table 4).

### 2.5. Genotypes and Serum Lipid Levels

The HDL-C levels were different between the AA and AC/CC genotypes in the controls (*p =* 0.038) but not in the CAD and IS patients (Table 5). The controls with an AC/CC genotype had an increased serum HDL-C level compared to the controls with an AA genotype.

## 3. Discussion

Despite therapeutic advances that control many risk factors, such as LDL-C, to levels lower than previously possible, death from cardiovascular disease continues to increase worldwide. Statins have reduced the risk of complications and death from cardiovascular causes by only approximately one third, leaving the remaining two thirds of patients unprotected [25]. Accordingly, the quest for pharmacologic agents that target other steps in atherogenesis has intensified in recent years. ACAT has been considered to be a promising drug target for therapeutic intervention against hyperlipidemia and atherosclerosis, and several clinical trials have tested the effects of ACAT inhibitors on the progression of atherosclerosis. However, whether *ACAT-1* inhibition will serve as an effective drug target for controlling atherosclerosis is under debate. Therefore, the genetic evidences for an association between the *ACAT-1* gene and atherosclerosis in humans still needs to been clarified.

The associations between *ACAT-1* gene polymorphisms and serum lipid levels were reported in several studies. Takao *et al*. [26] identified a missense variant (R526G) and a variant in the 5′ untranslated region (77G-A) in *ACAT-1*, and no significant association was detected between both SNPs and hyperlipidemia. However, plasma concentrations of HDL-C and ApoAI in subjects with the 77G-A variant were significantly higher than in hyperlipidemic subjects without the variant. In a previous study, we also showed that rs1044925 was associated with increased serum HDL-C only in hyperlipidemic subjects [24]. These data suggest that the *ACAT-1* variants may be associated with reduced ACAT-1 protein expression and influence cellular cholesterol efflux on hyperlipidemia, thereby playing an important role in atherosclerosis progression. In the general populations, the rs1044925 C allele is also associated with protective serum lipid profiles. In the Guangxi Bai Ku Yao population, serum lipid levels were obviously lower than those in Han Chinese. The female subjects with an AC/CC genotype had lower serum TC, LDL-C and ApoB than those with a common AA genotype [27]. Similar findings were also found in the healthy controls. Li *et al*. [28] showed that serum LDL-C and non-HDL-C levels were lower in the C allele carriers than in the C allele noncarriers. In the present study, we found that the C allele of rs1044925 was associated with a higher serum HDL-C level in healthy controls. Thus, we infer that rs1044925 is associated with a risk of CAD and IS. To our knowledge, the association between rs1044925 and the risk of atherosclerosis has not been reported.

In the present study, we showed that the frequencies of the AC/CC genotypes and C allele were associated with a decreased risk of CAD and IS. Multivariate analysis showed that known risk factors, such as BMI, diabetes, hypertension and hyperlipidemia, were independently associated with CAD and IS. Additionally, the rs1044925 AC/CC genotypes were associated with a decreased risk of CAD and IS after adjusting for potential confounding factors. In the stratified analysis, a decreased risk of CAD and IS in the subjects with an AC/CC genotype was mainly observed in males, nondrinkers and those with a BMI ≥ 24 kg/m^2^. A decreased risk of CAD in the subjects with an AC/CC genotype was also observed in nonsmokers. Foam cell formation is a well-established hallmark of early-stage atherosclerosis. The accumulation of CEs as abundant cytoplasmic lipid droplets, catalyzed by ACAT-1, causes macrophages to be foamy in appearance. A close connection between the foam cell appearance and ACAT-1 protein expression has been demonstrated in macrophages present in human atherosclerotic lesions [29]. Therefore, the possible mechanism by which *ACAT-1* gene variants protect against atherosclerosis is that inhibiting *ACAT-1* may directly or indirectly diminish foamy macrophage formation, thus further reducing the incidence of atherosclerosis. However, this protective effect against atherosclerosis depends on the cellular cholesterol efflux ability in reverse cholesterol transport, by which HDL is able to extract excess cholesterol from peripheral tissues and transfer this cholesterol to the liver for biliary excretion. In a series of studies using macrophages from *ACAT-1*-knockout mice, because of the impaired of ABCA1-mediated cholesterol efflux, the complete deletion of macrophage *ACAT-1* may lead to free cholesterol accumulation and subsequent cytotoxicity to macrophages, thereby, exacerbating atherosclerotic lesions [20–23]. In the present study, C allele carriers had an increased serum HDL-C level, suggesting that cholesterol efflux was not impaired when ACAT-1 activity was partially attenuated. A recent study [30] indicated that a selective *ACAT-1* inhibitor, K-604, may not induce free cholesterol accumulation but may accelerate cholesterol efflux from macrophages in an ApoE-independent manner, which can support the conclusion that the C allele of rs1044925 is associated with a decreased risk of CAD and IS. However, there were conflicting results between the *ACAT-1* deletion model and the present study. To explain these discrepancies, we must understand that the *ACAT-1*-deletion mice show a complete absence of ACAT-1 enzyme, a condition that does not occur in pharmacologic interventions on *ACAT-1* in animals or in human patients. Due to the complete absence of the ACAT-1 enzyme, the abundant accumulation of free cholesterol may be beyond the ability of intrinsic intracellular cholesterol efflux. Therefore, the mouse knockout model may be the most appropriate for demonstrating the detrimental effects of drug overdoses in animals or in humans. Partial inhibition of *ACAT-1* reduced atherosclerosis, effectively suggesting that *ACAT-1* inhibitors prevent atherosclerosis in a dose-dependent manner. In three clinical trials in humans, nonselective inhibition of ACAT had no protective role on atherosclerotic lesions but may actually promote atherosclerosis [14–16]. However, the dose of ACAT inhibitor in these clinical trials was derived from the results of animal experiments or *in vitro*, the lipid metabolism and lesion biology differ from those in humans. The negative results may be ascribed to inappropriate dosing. Therefore, new agents with moderate inhibition of *ACAT-1* need to be developed and studied in new clinical trials.

As multifactorial diseases, both CAD and IS shared common risk factors, such as hypertension, diabetes, dyslipidemia and metabolic syndrome [31], and the common pathophysiologic mechanisms is atherosclerosis [32]. Atherosclerosis is an extremely complex disease process with a number of important cellular contributors, including endothelial cells, smooth muscle cells, and immune cells (monocyte and T cells) [33–35]. Traditional risk factors lead to atherosclerosis by disrupting the function of these cells [35,36]. There have been many hypotheses proposed to explain the underlying mechanisms of the initiation, progression and rupture of atherosclerotic plaque. The key points of these hypotheses include the following: (i) lipoprotein retention; (ii) endothelial dysfunction; (iii) immune and inflammation response of the artery; (iv) vascular smooth muscle cell (VSMC) proliferation; (v) lipid absorption by macrophages and VSMCs and the formation of foam cells; and (vi) platelet activation and thrombosis [37,38]. Various epidemiological studies in families and twins have revealed a genetic component to CAD and IS risk in humans, the gene variants involved in these pathways of atherosclerosis could contribute to cardiovascular disease risk. From over 1300 publications of genetic studies, the CAD gene database [37] includes information on more than 300 candidate genes for CAD. While a single gene or gene region only explains a small part of the cardiovascular disease risk, more large association studies, including more target genes, are needed to assess the risk of cardiovascular disease.

There are two potential limitations to the present study. First, a number of patients with CAD and IS take anti-atherosclerotic drugs, such as statins, ACE inhibitors, beta blockers, and aspirin, before being enrolled in the study, whereas the controls did not take any drugs. The levels of TC and LDL-C were also lower in the patients with IS and CAD than in the healthy controls. However, the drug information was missing for some IS and CAD patients. Thus, when we detected the associations between the genotypes and serum lipid levels in IS and CAD patients, interference by drug therapy could not be analyzed. Second, although we found that the rs1044925 C allele was associated with a decreased risk of CAD and IS, we did not detect an association between the rs1044925 C allele and the ACAT enzyme activity, which is important for a functional evaluation of this SNP.

## 4. Materials and Methods

### 4.1. Cases and Controls

A total of 587 patients with CAD and 555 patients with IS were recruited from hospitalized patients in the First Affiliated Hospital, Guangxi Medical University. All of the enrolled CAD patients were evaluated by coronary angiography due to suspected CAD or unrelated conditions requiring angiographic evaluation; the coronary angiograms were analyzed by two experienced interventional cardiologists. CAD was defined as significant coronary stenosis (≥50%) in at least one of the three main coronary arteries or their major branches (branch diameter ≥ 2 mm). Subjects with congenital heart disease and type I diabetes mellitus were excluded. All of the enrolled IS patients received a strict neurological examination and brain magnetic resonance imaging. The diagnosis of IS was according to the International Classification of Diseases (9th Revision). Patients with a transient ischemic attack, embolic brain infarction, stroke caused by inflammatory disease, cardioembolic stroke, autoimmune disease, or serious chronic diseases were excluded from this study. Subjects with a past history of CAD were also excluded from the study [39].

A total of 588 healthy controls matched by age, gender, and geographical area were included. The controls were judged to be free of CAD and IS by questionnaires, medical history, and clinical examination. All individuals enrolled were from the Han population in Guangxi, China. A standard questionnaire was used to ascertain general information and medical history from all participants. The study protocol was approved by the Ethics Committee of the First Affiliated Hospital, Guangxi Medical University. Informed consent was obtained from all subjects after receiving a full explanation of the study.

### 4.2. Genotyping and Biochemical Analysis

All of the biochemical assays and genotyping in CAD and IS patients were performed after hospitalization, and all of the venous blood samples were obtained from the patients and controls after at least 12 h of fasting. Genomic DNA was isolated from peripheral blood leukocytes using the phenol-chloroform method [24,27]. Genotyping of rs1044925 was performed by polymerase chain reaction and restriction fragment length polymorphism (PCR-RFLP). PCR amplification was performed using 5′-TATATTAAGGGGATCAGAAGT-3′ and 5′-CCACCTAAAAACATACTACC-3′ as the forward and reverse primer pairs respectively. Each 20 μL PCR reaction mixture consisted of 1 μL of genomic DNA, 0.5 μL of each primer (10 pmol/L), 10 μL of 2× Taq PCR Mastermix (constituent: 20 mM Tris-HCl, pH 8.3, 100 mM KCl, 3 mM MgCl_2_, 0.1 U Taq Polymerase/μL, 500 μM dNTP each; Tiangen, Beijing, China), and 8 μL of ddH_2_O (DNase/RNase-free). After initial denaturizing at 95 °C for 5 min, the reaction mixture was subjected to 33 cycles of 45 s denaturation at 95 °C, 30 s annealing at 53 °C and extension 50 s at 72 °C, followed by a final 10 min extension at 72 °C. After electrophoresis on a 1.5% agarose gel with 0.5 μg/mL ethidium bromide, the amplification products were visualized under ultraviolet light. Then 5 U of RsaI restriction enzyme was added directly to the PCR products (5 μL) and digested at 37 °C overnight. After restriction enzyme digestion of the amplified DNA, genotypes were identified by electrophoresis on 1.5% agarose gels and visualized with ethidium-bromide staining ultraviolet illumination. The genotypes were scored by an experienced reader blinded to the epidemiological data and serum lipid levels. Six samples (AA, AC and CC genotypes in two; respectively) detected by the PCR-RFLP were also confirmed by direct sequencing. The PCR products were purified by low melting point gel electrophoresis and phenol extraction, and then the DNA sequences were analyzed in Shanghai Sangon Biological Engineering Technology & Services Co., Ltd., Shanghai, China. The levels of TC, TG, HDL-C, and LDL-C in the samples were determined by enzymatic methods with commercially available kits. Serum Apo AI and ApoB levels were detected by an immunoturbidimetric immunoassay using a commercial kit [24,27].

### 4.3. Diagnostic Criteria

The normal values for serum TC, TG, HDL-C, LDL-C, ApoAI, ApoB and ApoAI to ApoB ratio at our Clinical Science Experiment Center (Nanning, China) were 3.10–5.17, 0.56–1.70, 0.91–1.81, 2.70–3.20 mmol/L, 1.00–1.78, 0.63–1.14 g/L, and 1.00–2.50 respectively. Individuals with TC > 5.17 mmol/L and/or TG > 1.70 mmol/L were defined as hyperlipidemic. Hypertension was diagnosed according to the criteria from the 1999 World Health Organization-International Society of Hypertension Guidelines for the management of hypertension. The diagnostic criteria for being overweight and obesity were according to the Cooperative Meta-analysis Group of China Obesity Task Force (Beijing, China). Normal weight, overweight and obesity were defined as a BMI < 24, 24–28, and > 28 kg/m^2^ respectively [40]. Diabetes was defined as a fasting plasma glucose ≥7.0 mmol/L or 2 h postprandial plasma glucose ≥11.1 mmol/L or as having been previously diagnosed with diabetes and receiving therapy [41].

### 4.4. Statistical Analyses

All statistical analyses were performed using the statistical software package SPSS 13.0 (SPSS Inc., Chicago, IL, USA). A standard goodness-of-fit test was used to test the Hardy-Weinberg equilibrium. A chi-square analysis was used to evaluate the difference in genotype distribution and sex ratio between the groups. The general characteristics between the cases and controls were tested using Student’s unpaired *t*-test. The association between genotype and serum lipid parameters was tested by analysis of covariance (ANCOVA). Sex, age, BMI, blood pressure, alcohol consumption, and cigarette smoking were adjusted for in the statistical analysis. ORs and 95% CIs were calculated using conditional logistic regression. A two-tailed *p* value less than 0.05 was considered to be statistically significant.

## 5. Conclusions

The present study shows that the genotypic and allelic frequencies of rs1044925 were significantly different between the patients with CAD or IS and controls. Subjects with CC genotype or C allele were associated with a decreased risk of CAD in males, nondrinkers, nonsmokers and subjects with a BMI ≥ 24 kg/m^2^ and a decreased risk of IS in males, nondrinkers and subjects with a BMI ≥ 24 kg/m^2^. The AC/CC genotypes were also associated with increased serum HDL-C in the healthy controls. These results suggest that the rs1044925 C allele was associated with increased serum HDL-C in the healthy controls and with a decreased risk of CAD and IS.

## Figures and Tables

**Table 1.** General characteristics and serum lipid levels in the controls and patients.

Parameter	Control	CAD	IS	*P*1	*P*2
Number	588	587	555	–	–
Male/female	427/161	433/154	400/155	0.674	0.805
Age (years)	61.51 ± 10.85	62.25 ± 10.55	62.85 ± 12.33	0.236	0.052
Body mass index (kg/m^2^)	22.42 ± 2.85	24.03 ± 3.20	23.41 ± 3.52	<0.001	<0.001
Systolic blood pressure (mmHg)	130.45 ± 20.13	133.32 ± 23.56	147.74 ± 22.08	0.026	<0.001
Diastolic blood pressure (mmHg)	82.23 ± 13.24	79.37 ± 14.28	83.69 ± 12.77	<0.001	0.059
Pulse pressure (mmHg)	49.84 ± 15.06	53.49 ± 18.31	63.89 ± 18.25	<0.001	<0.001
Cigarette smoking (*n* (%))	258 (43.9)	274 (46.7)	224 (40.4)	0.335	0.253
Alcohol consumption (*n* (%))	267 (45.4)	159 (27.1)	166 (29.9)	<0.001	<0.001
Total cholesterol (mmol/L)	4.93 ± 1.04	4.54 ± 1.22	4.53 ± 1.14	<0.001	<0.001
Triglyceride (mmol/L)	1.41 ± 1.75	1.67 ± 1.13	1.68 ± 1.37	0.004	0.005
HDL-C (mmol/L)	1.88 ± 0.50	1.15 ± 0.34	1.23 ± 0.41	<0.001	<0.001
LDL-C (mmol/L)	2.74 ± 0.79	2.71 ± 1.03	2.68 ± 0.90	0.489	0.238
Apolipoprotein (Apo) AI (g/L)	1.41 ± 0.27	1.05 ± 0.55	1.03 ± 0.25	<0.001	<0.001
ApoB (g/L)	0.89 ± 0.21	0.91 ± 0.27	0.89 ± 0.25	0.376	0.992
ApoAI/ApoB	1.67 ± 0.59	1.25 ± 0.82	1.19 ± 0.60	<0.001	<0.001

CAD, coronary artery disease; IS, ischemic stroke; HDL-C, high-density lipoprotein cholesterol; LDL-C, low-density lipoprotein cholesterol; *P*1: comparison of CAD and control; *P*2: comparison of IS and control.

**Table 2.** Genotypic and allelic frequencies and the risk of coronary artery disease (CAD) and ischemic stroke (IS).

Genotype or allele	Control (*n* (%))	CAD (*n* (%))	IS (*n* (%))	CAD		IS	

	*n* = 588	*n* = 587	*n* = 555	OR (95% CI)	*p*	OR (95% CI)	*p*
AA	412 (70.1)	452 (77.0)	424 (76.4)	1		1	
AC	166 (28.2)	123 (21.0)	125 (22.5)	0.67 (0.50–0.91)	0.009	0.72 (0.54–0.95)	0.021
CC	10 (1.7)	12 (2.0)	6 (1.1)	0.95 (0.36–2.53)	0.921	0.56 (0.20–1.57)	0.269
χ^2^		8.431	6.001				
*P*		0.015	0.050				
AA	412 (70.1)	452 (77.0)	424 (76.4)	1		1	
AC + CC	176 (29.9)	135 (23.0)	131 (23.6)	0.69 (0.52–0.93)	0.014	0.71 (0.54–0.93)	0.014
χ^2^		7.256	5.820				
*P*		0.007	0.016				
A	990 (84.2)	1027 (87.5)	973 (87.7)	1		1	
C	186 (15.8)	147 (12.5)	137 (12.3)	0.76 (0.60–0.96)	0.022	0.75 (0.59–0.95)	0.017
χ^2^		5.245	5.680				
*P*		0.022	0.017				

Adjusted for sex, age, smoking, drinking, BMI, diabetes, hypertension, hyperlipidia. CAD, coronary artery disease; IS, ischemic stroke.

**Table 3.** The risk of rs1044925 for CAD and IS according to body mass index (BMI), gender, smoking and drinking.

Factors	Genotype	CAD			IS		

		OR (95% CI)	*p*	*p_interaction_*	OR (95% CI)	*p*	*p_interaction_*
BMI							

<24 kg/m^2^	AA	1			1		
	AC + CC	0.79 (0.54–1.14)	0.208		0.82 (0.58–1.14)	0.238	
≥24 kg/m^2^	AA	1		0.507			0.221
	AC + CC	0.57 (0.36–0.91)	0.018		0.53 (0.34–0.84)	0.007	

Gender							

Male	AA	1			1		
	AC + CC	0.71 (0.51–1.00)	0.050		0.69 (0.50–0.95)	0.024	
Female	AA	1		0.777	1		0.946
	AC + CC	0.67 (0.37–1.20)	0.178		0.75 (0.44–1.29)	0.295	

Smoking							

Nonsmoker	AA	1			1		
	AC + CC	0.63 (0.42–0.94)	0.023		0.73 (0.52–1.04)	0.078	
Smoker	AA	1		0.459	1		0.980
	AC + CC	0.79 (0.51–1.22)	0.288		0.71 (0.42–1.09)	0.112	

Drinking							

Nondrinker	AA	1			1		
	AC + CC	0.66 (0.47–0.93)	0.017		0.68 (0.49–0.95)	0.024	
Drinker	AA	1		0.831	1		0.709
	AC + CC	0.75 (0.44–1.28)	0.291		0.75 (0.47–1.21)	0.242	

CAD, coronary artery disease; IS, ischemic stroke.

**Table 4.** The relative risk factors for CAD and IS.

Relative Factors	CAD		IS	

	OR (95% CI)	*p*	OR (95% CI)	*p*
Nonsmoking	1		1	
Smoking	1.39 (1.06–1.82)	0.016	1.05 (0.80–1.38)	0.724
Nondrinking	1		1	
Drinking	0.29 (0.22–0.39)	<0.001	0.41 (0.31–0.55)	<0.001
BMI < 24 kg/m^2^	1		1	
BMI ≥ 24 kg/m^2^	2.37 (1.82–3.08)	<0.001	1.60 (1.22–2.88)	0.001
rs1044925 AA	1		1	
rs1044925 AC/CC	0.71 (0.53–0.95)	0.020	0.66 (0.50–0.89)	0.005
Non-diabetes	1		1	
Diabetes	4.50 (3.05–6.65)	<0.001	2.68 (1.72–3.92)	<0.001
Normotensive	1		1	
Hypertension	2.18 (1.72–2.76)	<0.001	3.33 (2.57–4.32)	<0.001
Normal blood lipids	1		1	
Hyperlipidemia	1.67 (1.32–2.11)	<0.001	2.03 (1.57–2.55)	<0.001

CAD, coronary artery disease; IS, ischemic stroke.

**Table 5.** Association between rs1044925 and serum lipid levels in controls and CAD and IS patients.

Genotype	*n*	TC (mmol/L)	TG (mmol/L)	HDL-C (mmol/L)	LDL-C (mmol/L)	ApoAI (g/L)	ApoB (g/L)	ApoAI/ApoB
Control								

AA	412	4.89 ± 0.92	1.29 ± 1.22	1.85 ± 0.48	2.76 ± 0.78	1.40 ± 0.28	0.89 ± 0.21	1.66 ± 0.63
AC/CC	176	4.97 ± 1.24	1.35 ± 2.64	1.94 ± 0.55	2.71 ± 0.82	1.42 ± 0.25	0.89 ± 0.22	1.68 ± 0.47
*F*	–	0.959	0.590	4.311	0.616	0.793	0.000	0.113
*p*	–	0.328	0.443	0.038	0.433	0.373	0.996	0.737

CAD								

AA	452	4.51 ± 1.18	1.68 ± 1.18	1.14 ± 0.35	2.68 ± 1.00	1.03 ± 0.37	0.90 ± 0.26	1.21 ± 0.58
AC/CC	135	4.58 ± 1.34	1.58 ± 1.00	1.18 ± 0.33	2.73 ± 1.08	1.11 ± 0.93	0.88 ± 0.30	1.37 ± 1.34
*F*	–	0.418	1.109	0.966	0.256	2.190	1.728	3.727
*p*	–	0.518	0.293	0.326	0.613	0.130	0.189	0.054

IS								

AA	424	4.53 ± 1.20	1.68 ± 1.47	1.24 ± 0.43	2.69 ± 0.94	1.03 ± 0.23	0.89 ± 0.25	1.19 ± 0.64
AC/CC	131	4.58 ± 0.95	1.72 ± 1.08	1.17 ± 0.31	2.73 ± 0.76	1.00 ± 0.18	0.92 ± 0.23	1.14 ± 1.41
*F*	–	0.294	0.098	3.351	0.241	1.714	2.098	0.487
*p*	–	0.588	0.755	0.068	0.619	0.191	0.148	0.486

Adjusted for sex, age, smoking, drinking, BMI, diabetes, hypertension, hyperlipidia. TC, total cholesterol; TG, triglyceride; HDL-C, high-density lipoprotein cholesterol; LDL-C, low-density lipoprotein cholesterol; ApoAI, apolipoprotein AI; ApoB, apolipoprotein B.
